# Supplementary material for: Identification of photoperiod-induced specific miRNAs in the adrenal glands of Sunite sheep (Ovis aries)
Source: Front Vet Sci. 2022 Jul 22;9:888207. doi: 10.3389/fvets.2022.888207 (PMC9354845; doi:10.3389/fvets.2022.888207)
Supplement: Supplementary file 1 [file Data_Sheet_1.ZIP › Supplementary file/miRDeep2 file.zip!/miRDeep2 file/Know_Structure/SPLP42A2/oar-mir-181a-2.pdf]

miRBase precursor	: oar-mir-181a-2
Total read count	: 16185
oar-miR-181a read count	: 16066
remaining reads	: 119

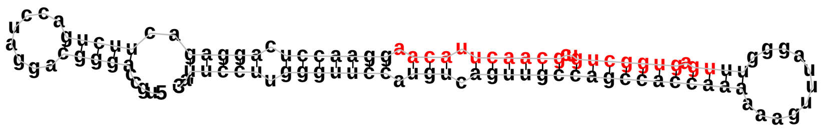

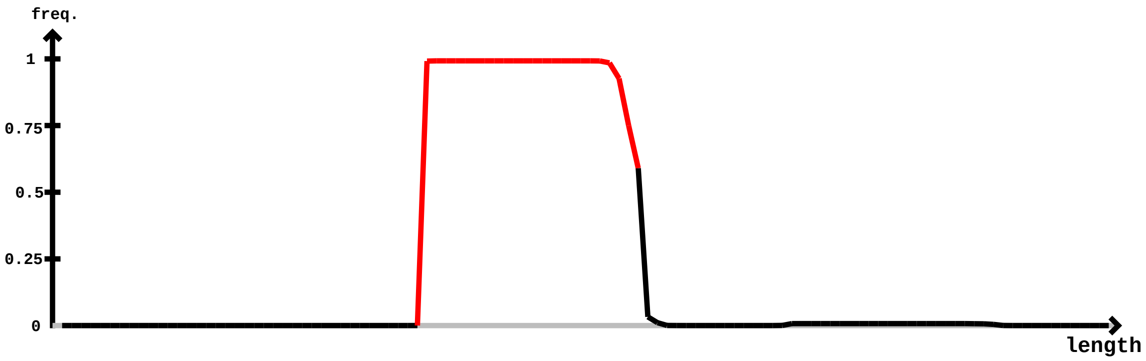

oar-miR-181a

```
5'- ugccagggcaggauccagucuucagaggacuccaaggaacauucaacgcugucggugaguuugggauuugaaaaaaccaccgaccguugacuguaccuuggguuccuua   -3'    exp
    ....(((((........)))))..(((((.(((((((.(((.((((((..(((((((.((((...........)))))))))))))))).))).))))))).))))).    reads      mm         sample
    ....................................gaacauucaacgcugucggugag..................................................    1          0          seq
    .....................................aacauucaacgcugucgg......................................................    7          0          seq
    .....................................aacauucaacgcugucggu.....................................................    115        0          seq
    .....................................aacauucaacgcugucggug....................................................    949        0          seq
    .....................................aacauucaacgcugucgguga...................................................    2857       0          seq
    .....................................aacauucaacgcugucggugag..................................................    2588       0          seq
    .....................................aacauucaacgcugucggugagu.................................................    9006       0          seq
    .....................................aacauucaacgcugucggugaguu................................................    354        0          seq
    .....................................aacauucaacgcugucggugaguuu...............................................    167        0          seq
    .....................................aacauucaacgcugucggugaguuug..............................................    6          0          seq
    .....................................aacauucaacgcugucggugaguuugg.............................................    1          0          seq
    ......................................acauucaacgcugucggu.....................................................    1          0          seq
    ......................................acauucaacgcugucggugag..................................................    1          0          seq
    ......................................acauucaacgcugucggugagu.................................................    11         0          seq
    ......................................acauucaacgcugucggugaguu................................................    1          0          seq
    ...........................................caacgcugucggugagu.................................................    1          0          seq
    ..........................................................................aaccaccgaccguugacugu...............    1          0          seq
    ..........................................................................aaccaccgaccguugacuguac.............    6          0          seq
    ..........................................................................aaccaccgaccguugacuguacc............    1          0          seq
    ...........................................................................accaccgaccguugacugu...............    7          0          seq
    ...........................................................................accaccgaccguugacugua..............    5          0          seq
    ...........................................................................accaccgaccguugacuguac.............    28         0          seq
    ...........................................................................accaccgaccguugacuguacc............    65         0          seq
    ...........................................................................accaccgaccguugacuguaccu...........    5          0          seq
    ............................................................................ccaccgaccguugacugua..............    1          0          seq
```
